# Editorial: Connections to Membrane Trafficking Where You Least Expect Them: Diseases, Dynamics, Diet and Distance

**DOI:** 10.3389/fcell.2019.00327

**Published:** 2019-12-06

**Authors:** Sarita Hebbar, Elisabeth Knust, Guillaume Thibault, Rachel Susan Kraut

**Affiliations:** ^1^Max Planck Institute of Molecular Cell Biology and Genetics, Dresden, Germany; ^2^Lipid Regulation and Cell Stress Research Group, School of Biological Sciences, Nanyang Technological University, Singapore, Singapore; ^3^Institute of Molecular and Cell Biology, A^*^STAR, Singapore, Singapore

**Keywords:** vesicles, membrane traffic, lipids, membrane dynamics, disease models

Membranous compartments within cells form not only an aesthetically interesting, dynamical three-dimensional structure—they also present a conundrum: how do these exotic vesicular, tubular, and pancake-like shapes meld into each other and communicate, yet maintain distinct identities and morphologies, in addition to steering the trafficking and precise targeting of their cargoes? How are their shapes, compositions, and positions in the cell controlled—all of which seem to be of prime importance to their functions? Perhaps most mysteriously of all, how does vesicle trafficking impinge upon spatially (and conceptually) distant downstream processes such as morphology and metabolism?

Defects in membrane trafficking can be disastrous for the cell and for the organism. Diseases as diverse as Parkinson's, diabetes, metabolic syndrome, and cancer are known to have at least some causal component, if not a central origin, related to vesicle trafficking and/or lipid metabolic control. An expanding array of possible causes and consequences of faulty trafficking among vesicular compartments is being cataloged, with ever more surprising connections being discovered.

Vesicular organelles were once thought to function as more or less independent entities, but cellular processes demand an interplay between different compartments. One way in which different membrane-bound compartments interact is via physical contact. Although previously underappreciated, now with the help of the newest wave of high-resolution imaging techniques, vesicular organelles have been observed in the act of intimate contact and lipid exchange. Endoplasmic reticulum (ER)-mitochondrial contacts were an early example of such conduits of transport, but now nearly all possible pairings have been witnessed and their purposes described. The review by Joshi and Cohen in this collection comprehensively summarizes the links between two intracellular metabolic hubs: lipid droplets (LD) and peroxisomes. LD and peroxisomes are tethered by interactions between spastin and ABCD1, but this review further elaborates on how these two compartments are also linked by their common origin in the same ER sub-domains.

Interaction of membrane-bound compartments also comes into play in the degradation of toxic, misfolded, or otherwise harmful proteins. For example, autophagy-based degradation is almost entirely dependent on the prerequisite of membrane transfer/contact starting from its biogenesis. Two research papers in this collection investigate the mechanisms underlying the relevance of membrane bound processes in degradation of protein and metabolite build-up (Jacomin et al.; Sim et al.).

Other functions mediated by vesicular membranes are less obvious. For example, one area that will surely break into this realm of research in a big way is the influence of metabolic factors like diet and oxidative stress. Several papers in this issue deal with this topic: Trautenberg et al. also related elements of the diet to potentially protective or degenerative intracellular signaling pathways, the relevant lipid cues derived from stationary *S. cerevisiae* activating Akt/PKB isoforms through the accumulation of PIP_3_.

One of the ways in which dietary lipids could affect trafficking is by changing the deformability and bending of membranes. This is a likely scenario, since dietary lipids alter the composition of cellular membranes, after a long journey through the digestive system and metabolism (Clandinin et al., 1983). Differences in physical and biochemical structure in turn affect both vesiculation (Pinot et al., 2014), and susceptibility to ROS-induced peroxidation. In pursuit of understanding the physical effects that could mediate these changes, Tyler et al. examined the effects of cis vs. trans fatty acids on the properties of membranes using X-ray diffraction and membrane fluctuation analysis.

Among the topics in this collection, we encounter innovative methods that are used in different model organisms to approach aspects of ROS- and free-radical mediated damage to neurons. For example, Jacomin et al. apply X-ray synchrotron spectromicroscopy for the first time to study the effects of age and autophagy on iron accumulation in the fly brain. Age, autophagy, and iron are factors associated with degeneration, partly via oxidative stress arising from non-degraded mitochondria or the damage done by the catalysis of free radical reactions. The use of this unconventional approach enabled the authors to flag Fe-S complexes in mitochondria, rather than total levels of Fe in the brain, as a likely culprit. Beaudoin-Chabot et al. whose study similarly dealt with the effects of oxidative stress-inducing conditions showed, by feeding deuterated polyunsaturated fatty acids (dPUFAs) to *C. elegans* worms, that this diet could protect the animals against the deleterious effects of peroxidated lipids, extending the lifespan. The mechanism they propose helps to solve the long-standing problem of why antioxidants are frustratingly ineffective in combating degenerative conditions. In an overview of the involvement of another stress- and degeneration-related phenomenon, ER stress, Chadwick and Lajoie describe its interplay with lipids, autophagy, and aging, touching on many of the factors mentioned above.

Membrane biophysics and dynamics are important determinants of vesicular behavior and have accordingly been a subject of keen interest. Many questions concern the influence of lipid composition, curvature, and specific lipid species on membrane dynamics, and how these either control or are controlled by membrane sub-domains. For instance, lipid mediators are surely involved in the corralling of receptors into an array of specific domains at the plasma membrane surface, which in turn affect endocytic uptake, and thus function. Phenomena like this are described by Busto and Wedlich-Söldner in their review of the domain segregation of nutrient transporters in yeast.

In addition to lateral segregation of different classes of membrane lipids influencing endocytic budding, the sorting of particular signaling species, like phosphatidic acid (PA), to different membrane locales can also regulate intracellular membrane transport and the activities of associated proteins. Thakur et al., provide a cell biological, analytical, and biochemical perspective on PA structure, metabolism, and its segregation into distinct functional pools. Interestingly this segregation is crucial for the signaling functioning of PA with consequences for tissue development, health, and physiology.

There is a vast diversity of lipid species in eukaryotes (Wenk, 2010). How this diversity contributes to specific membrane trafficking routes is unanswered. Corollary to this is the question of how the spatial distribution of the lipid species in the different membrane-bound compartments is achieved, especially (i) within organelles and (ii) when they exchange membranes during transport.

Another recurring theme in the collection is the surprising role of adaptors or accessory proteins in different vesicular systems (see Lurick et al., 2018 for review) in neuronal morphology, e.g., early-to-late endocytic trafficking and autophagy: Harish et al., for example show that dMon1, a Rab7 exchange factor involved in conversion of early to late endosomes in the fly, puts the brakes on dendritic branching of neurons. This was mediated, not as expected, through “passive” interference with lysosomal trafficking, but rather through a Rab11 recycling pathway at the membrane. Sim et al. used live primary neurons, also in the fly, to show that a BEACH domain protein, known previously only as a Rab11-interacting autophagic adaptor, not only actively moves around between early and late autophagic vesicles depending on the stressor (starvation or protein aggregates) but when overexpressed greatly increases the numbers of mature autophagosomes. Coincidentally, like dMon1, the protein was originally identified because of an effect on neuronal branching patterns (Kraut et al., 2001). Here, we repeatedly see trafficking systems regulated by Rab proteins and their adaptors or associates, that also control morphological processes, seemingly far away from the vesicular events themselves. These reports together with the literature open new questions on the role of guanine nucleotide exchange factors (GEFs) in trafficking. What are the different guanine nucleotide exchange factors (GEFs) and GTPase-activating proteins (GAPS) that coordinate different transport routes? How their perturbation affects cargo and the transport routes? Is this specific to cell-types?

The questions abound—as do the methods and model systems used to address these. An exciting sampling of some of these will be presented in this Research Topic on connections to membrane trafficking.

## Author Contributions

All authors listed have made a substantial, direct and intellectual contribution to the work, and approved it for publication.

### Conflict of Interest

The authors declare that the research was conducted in the absence of any commercial or financial relationships that could be construed as a potential conflict of interest.
